# LIFE SATISFACTION OF OLDER ADULTS: IMPACT OF LEISURE PARTICIPATION AND REGIONAL CHARACTERISTICS

**DOI:** 10.1093/geroni/igae098.2779

**Published:** 2024-12-31

**Authors:** Rokbit Lee, Jinmoo Heo, Inheok Lee, May Kim, Sua Im

**Affiliations:** Yonsei University, Seoul, Republic of Korea; Yonsei University, Seoul, Republic of Korea; University of Georgia, Athens, Georgia, United States; Korea University, Seoul, Republic of Korea; Yonsei University, Seoul, Republic of Korea

## Abstract

As Korea is transitioning into an aged-society, improving well-being of older adults has become important, and literature has demonstrated various individual and societal factors that impact their well-being. The purpose of this study is to explore the role of individual and regional factors that are associated with older adults’life satisfaction. Specifically, we used leisure participation as individual factor and regional characteristics include perceived stress and volunteer participation rate. Data were drawn from the 2020 National Survey of Korean Elderly and Statistics Korea. As the individual data were nested in regions (cities and provinces), we used hierarchical linear modeling to analyze the data. Findings show that volunteering, traveling, and participating in club activities were positively related to life satisfaction of older adults. The relationship observed between older adults’ participation in travel, club activities, volunteering, and life satisfaction suggests that engaging in productive leisure activities is a crucial aspect of daily life of older adults. At regional level, perceived stress level was negatively associated with life satisfaction whereas volunteer participation rate was positively related to life satisfaction. The negative correlation between older adults’ perceived local stress and life satisfaction, and the positive correlation between local social volunteer participation rate and life satisfaction suggest that stress perceived by individuals due to their residential area or surrounding environment has a negative impact on their lives. A statistically significant cross-level interaction was observed indicating that the impact of volunteer participation rate on life satisfaction was stronger in regions with higher levels of perceived stress rate.

